# Ultrasound-Assisted Extraction, Centrifugation and Ultrafiltration: Multistage Process for Polyphenol Recovery from Purple Sweet Potatoes

**DOI:** 10.3390/molecules21111584

**Published:** 2016-11-20

**Authors:** Zhenzhou Zhu, Tian Jiang, Jingren He, Francisco J. Barba, Giancarlo Cravotto, Mohamed Koubaa

**Affiliations:** 1 College of Food Science and Engineering, Wuhan Polytechnic University, Wuhan 430023, China; zhenzhouzhu@126.com (Z.Z.); 13476183768@163.com (T.J.); 2 Nutrition and Food Science Area, Preventive Medicine and Public Health, Food Science, Toxicology and Forensic Medicine Department, Faculty of Pharmacy, Universitat de València, Avda. Vicent Andrés Estellés, s/n, 46100 Burjassot, València, Spain; 3 Dipartimento di Scienza e Tecnologia del Farmaco, University of Turin, Via P. Giuria 9, 10125 Turin, Italy; giancarlo.cravotto@unito.it; 4 Centre de Recherche de Royallieu, Laboratoire Transformations Intégrées de la Matière Renouvelable (UTC/ESCOM, EA 4297 TIMR), Université de Technologie de Compiègne, CS 60319, 60203 Compiègne CEDEX, France; m.koubaa@escom.fr

**Keywords:** purple sweet potato, ultrasound, polyphenols, filtration, centrifugation

## Abstract

This work provides an evaluation of an ultrasound-assisted, combined extraction, centrifugation and ultrafiltration process for the optimal recovery of polyphenols. A purple sweet potato (PSP) extract has been obtained using ultrasonic circulating extraction equipment at a power of 840 W, a frequency of 59 kHz and using water as solvent. Extract ultrafiltration, using polyethersulfone (PES), was carried out for the recovery of polyphenol, protein and anthocyanin. Pre-treatment, via the centrifugation of purple sweet potato extract at 2500 rpm over 6 min, led to better polyphenol recovery, with satisfactory protein removal (reused for future purposes), than PSP extract filtration without centrifugation. Results showed that anthocyanin was efficiently recovered (99%) from permeate. The exponential model fit well with the experimental ultrafiltration data and led to the calculation of the membrane’s fouling coefficient. The optimization of centrifugation conditions showed that, at a centrifugation speed of 4000 rpm (1195× *g*) and duration of 7.74 min, the optimized polyphenol recovery and fouling coefficient were 34.5% and 29.5 m^−1^, respectively. The removal of proteins in the centrifugation process means that most of the anthocyanin content (90%) remained after filtration. No significant differences in the intensities of the HPLC-DAD-ESI-MS^2^ peaks were found in the samples taken before and after centrifugation for the main anthocyanins; peonidin-3-feruloylsophoroside-5-glucoside, peonidin-3-caffeoyl-*p*-hydroxybenzoylsophoroside-5-glucoside, and peonidin-3-caffeoyl-feruloyl sophoroside-5-glucoside. This proves that centrifugation is an efficient method for protein removal without anthocyanin loss. This study considers this process an ultrasound-assisted extraction-centrifugation-ultrafiltration for purple sweet potato valorization in “green” technology.

## 1. Introduction

The use of synthetic pigments is becoming ever more restricted in a number of countries due to concerns over their association with diseases, including cancer and heart disease [1]. These restrictions have led both food researchers and food industries to replace them, in food products, with natural molecules, especially polyphenols, which display numerous benefits (e.g., antioxidant activity, hepato-protectant, etc.) [2,3,4].

Purple sweet potatoes (PSP) have been studied as a potential source of polyphenols, which could provide important applications in functional foods [3,5]. In fact, a number of conventional and non-conventional methodologies have been used for their extraction. These methods include ultrasound-assisted extraction (UAE), which is considered to be one of the most economic and efficient techniques for the recovery of polyphenols and other valuable compounds from PSP [6,7].

It is widely believed that the cavitation effect caused by ultrasound waves enhances cell disruption, solvent penetration and mass transfer [8,9,10,11], thus intensifying target molecular extraction. In a recent work, UAE has been successfully demonstrated to work in green extraction of polyphenols from PSP, using water as solvent [4]. Despite the unsatisfying polyphenol yields recovered under ultrasound conditions, advanced treatment may lead to improved denaturation of PSP tissues and the enhanced release of target molecules.

The feasibility of using ultrafiltration for polyphenol recovery and protein removal (major impurity) from PSP extracts has been recently investigated [12]. However, although the polyphenol content in the permeate increased, the filtration flux decreased because of membrane fouling caused by the accumulation and deposition of particles on the membrane surface, thus clogging the filtration pores. Membrane fouling is one of the major problems encountered in the food industry when using similar technologies. This fact limits the productivity of the process and means that additional maintenance steps are required. Controlling membrane fouling is a key to overcoming this problem and therefore achieving the efficient recovery and purification of target molecules (i.e., polyphenols). Fouling control has always been performed by preventing the formation of fouling elements using trans-membrane pressure, solute size and physicochemical properties as well as foulant and membrane characteristics [13].

The pre-treatment of feed juice is one of the most effective membrane fouling elimination technologies [13,14]. Centrifugation is an important juice pre-treatment method that has a great effect on membrane fouling and filtration kinetics by removing suspended particles [15].

Moreover, the biological activity and retention of polyphenols differ according to their chemical structure and the affinity of these compounds to the purification systems. It is thus necessary to study polyphenol profiles using innovative analytical tools, such as high performance liquid chromatography (HPLC) (with diode array detector (DAD)) coupled to mass spectrometry (MS) (with electro-spray ionization interface (ESI)) (HPLC-DAD-ESI-MS^2^). A detailed investigation of the centrifugation conditions, such as the centrifugation speed and duration, in PSP extraction ultrafiltration (UF) performance is therefore of paramount importance if polyphenols are to be recovered at higher productivities.

In the present study, polyphenols have been extracted from PSP using ultrasonic circulating extraction equipment. The extract was then pre-treated with centrifugation and separated with ultrafiltration. Filtration behavior and permeate quality have been analyzed. The impact of centrifugation conditions on polyphenol recovery and filterability (membrane fouling) have been investigated and furnished optimal centrifugation conditions. The HPLC-DAD-ESI-MS^2^ polyphenol profiles have also been investigated before and after the centrifugation and filtration processes.

## 2. Results and Discussion

### 2.1. Impact of Centrifugation Pre-Treatment on UF Efficiency

**Figure 1:**
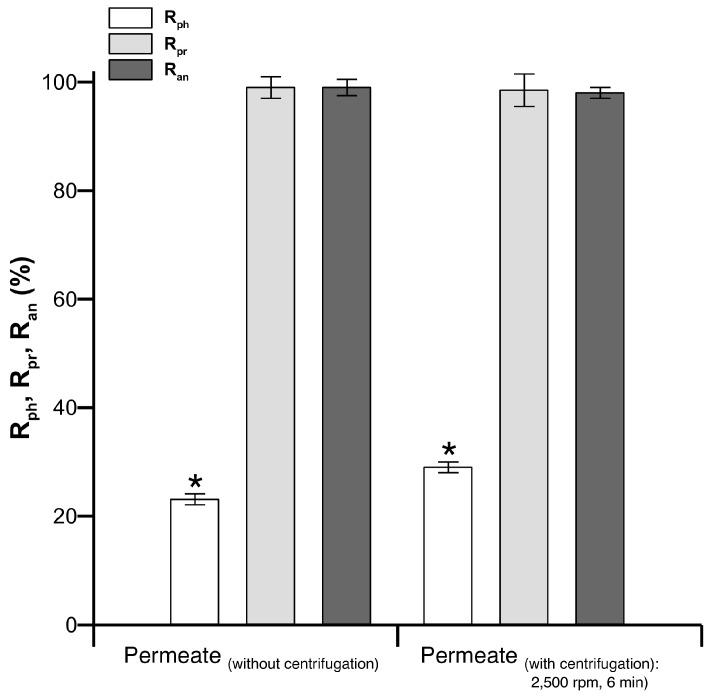
Polyphenol, protein and anthocyanin recovery after the ultrafiltration of purple sweet potato (PSP) extracts, both without centrifugation and with centrifugation at 2500 rpm during 6 min. Error bars correspond to standard deviations. Significant differences were marked with asterisk.

The contents of polyphenols, anthocyanins and proteins in the PSP extract were found to be 1.30 ± 0.01 mg/g, 0.13 ± 0.00 mg/g and 40.0 ± 0.01 mg/g. The efficiencies of UF in PSP extract purification, with and without centrifugation pre-treatment, are presented in Figure 1. Results show that better PSP juice polyphenol recovery (R_ph_) (29% ± 1%) was observed after centrifugation at 2500 rpm for 6 min, whereas the raw PSP extract provided lower recovery (23% ± 1%). This result demonstrates that centrifugation pre-treatment can improve the recovery of polyphenols from a PSP extract. This result could be attributed to the removal of fine particles, which may enhance fouling propensity during the filtration process. These fine particles are mainly composed of colloid aggregates, known to be highly involved in membrane fouling [16]. Their accumulation on the surface of the membrane may result in the build-up of a tight network with higher strength, leading to more polyphenol retention. The protein removal results (≈99%) show that centrifugation pre-treatment did not affect protein elimination via the UF process, implying that the retention of proteins is most likely dominated by membrane pore size, not the fouling layer. Moreover, satisfactory anthocyanin recovery was confirmed, proving that centrifugation and ultrafiltration did not affect the permeation of anthocyanin.

**Figure 2:**
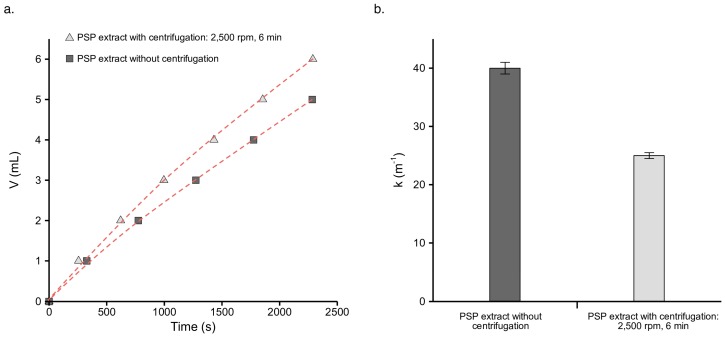
(**a**) Permeate volume versus filtration time. The points represent the experimental data and the dashed lines represent the fitted results after applying Equation (2); and (**b**) fouling coefficient (k) for the filtration of the PSP extract without centrifugation and PSP juice with centrifugation (2500 rpm, 6 min). Error bars correspond to standard deviations.

An analysis of permeate quality highlighted the improvement that centrifugation pre-treatment had on the recovery of polyphenols. The effect of centrifugation on filtration behavior and membrane fouling was also investigated (Figure 2). The filtration behavior of PSP juice, obtained after centrifugation at 2500 rpm over 6 min, was clearly better than that of the PSP extracted without centrifugation. These results confirm that centrifugation can improve both the recovery of polyphenols and filtration behavior. Under the same filtration operating conditions (trans-membrane pressure (TMP), rotation speed, membrane pore size) better filtration behavior is generally obtained when less fouling occurs. In order to explain the drop in membrane fouling generated by centrifugation, the exponential model given by Equation (6) was applied to fit the experimental data (solid points in Figure 2a), and the fitting curves are shown as dashed lines. This model had successfully been used previously for the investigation of chicory juice filtration using an Amicon 8200 (Millipore, Bedford, MA, USA) [13]. Similarly, the fouling coefficients for the filtration of PSP exact, with and without centrifugation, were calculated. The results presented in Figure 2b show that the fouling coefficient of PSP juice decreased after centrifugation, as compared to PSP exact obtained without centrifugation.

**Figure 3:**
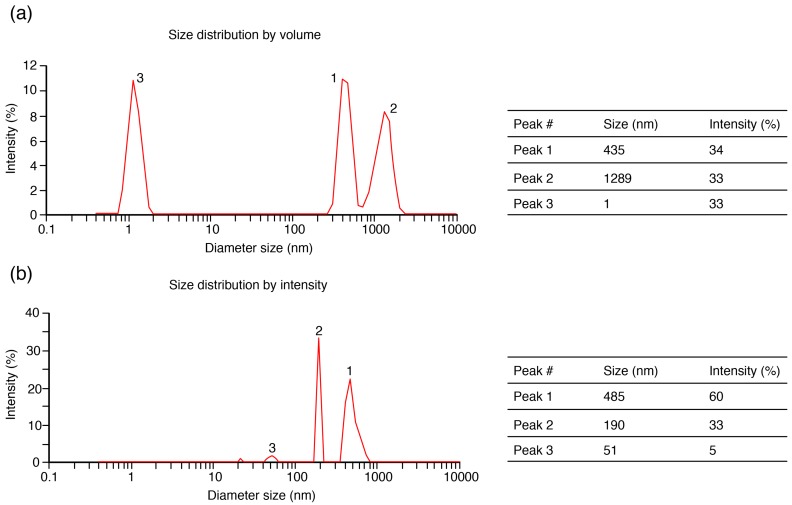
Average size distributions for particles in PSP extracts before centrifugation (**a**) and after centrifugation (**b**).

The results highlight the improved filterability of PSP juice after centrifugation pre-treatment, which removes particles in the PSP extract. The particle size distributions of both feeds were then analyzed (Figure 3) to verify the above assumptions. Before centrifugation, particle sizes were mainly around 1, 435, and 1289 nm, whereas, after centrifugation, the particle sizes were 195 and 485 nm. The complex composition of the suspension for PSP juice before centrifugation resulted in more serious fouling and declined flux.

### 2.2. Optimization of the Centrifugation Conditions

Despite the positive effect that centrifugation has on the recovery of polyphenols and filtration kinetics, which has been supported by the above discussion, literature studies have also shown that centrifugation conditions have a significant effect on separation and filtration performance [15,17,18]. Detailed investigation and optimization of the centrifugation conditions (centrifugation speed and time) were thus carried out.

In order to obtain a well-fitted model, the linear regression coefficients for the model and the test for the lack of fit were determined. The results of the quadratic response surface model for polyphenol recovery and the fouling coefficient are presented in Table 1 in the form of an analysis of variance (ANOVA). The response surface methodology (RSM) model *p*-value for the recovery of polyphenols was 0.0349, meaning significance at 95% confidence. The *p*-value for the lack of fit was 0.741, which indicates that it is not significantly different from the pure error. The same analysis can also be applied to the fouling coefficient according to the ANOVA results presented in Table 1. The response surface models developed for all the response variables can therefore be stated to be adequate.

Equations (1) and (2) give the equations in terms of coded variables achieved by applying multiple regression analyses to the experimental data:

(1) $$R_{\mathrm{ph}}=28.68+2.42{\;x}_{1}-0.19{\;x}_{2}+2.75{\;x}_{1}x_{2}+1.81{\;x}_{1}^{2}+2.46{\;x}_{2}^{2},$$

(2) $$k=26.95-1.98{\;x}_{1}+0.5{\;x}_{2}+1.42{\;x}_{1}x_{2}+2.72{\;x}_{1}^{2}+5.25{\;x}_{2}^{2},$$

where x_1_ and x_2_ represent the coded variables of TMP and shear rate, respectively.

The models in terms of actual variables obtained from Equations (1) and (2) and the actual values are presented in Equations (3) and (4):

(3) $$R_{\mathrm{ph}}=42.38-5.16\;10^{-3}X_{1}-3.04{\;X}_{2}+4.58\;10^{-4}X_{1}X_{2}+8.04\;10^{-7}X_{1}^{2}+0.15X_{2}^{2},$$

(4) $$k=52.43-8.79\;10^{-3}X_{1}-4.41{\;X}_{2}+2.37\;10^{-4}X_{1}X_{2}+1.21\;10^{-6}X_{1}^{2}+0.33{\;X}_{2}^{2}.$$

The quadratic response models given by Equations (3) and (4) can be used to predict the polyphenol recovery and fouling coefficients within the limits of the experimental domain.

**Figure 4:**
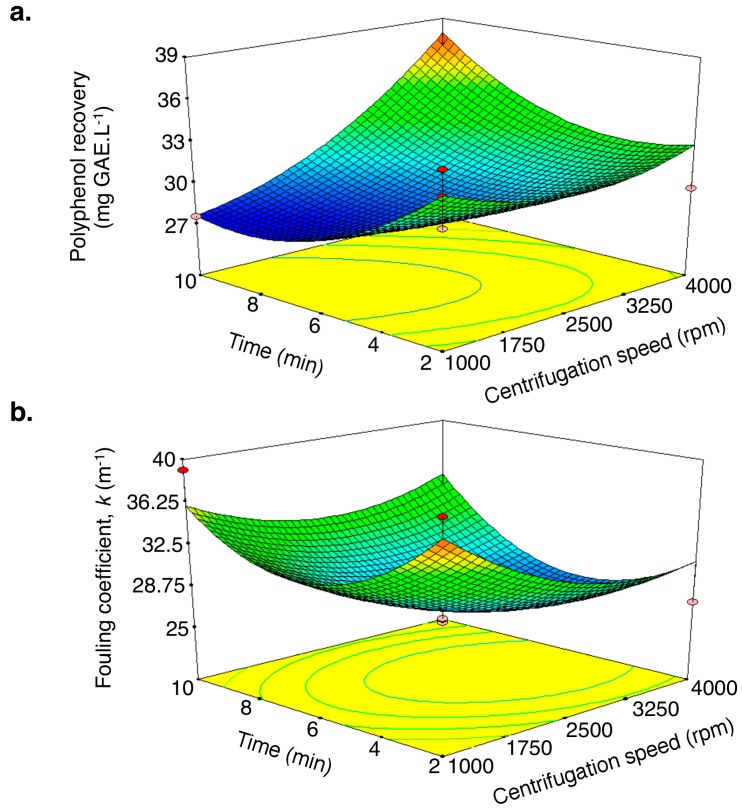
Surface response of polyphenol recovery (**a**) and fouling coefficient (**b**) as a function of centrifugation speed and time.

The combined effect of the centrifugation speed and time on polyphenol recovery is shown in Figure 4a,b. As expected, increasing the centrifugation speed led to the highest polyphenol recovery values and to less fouling, while the influence of centrifugation time was more complex. In fact, polyphenol recovery decreased with increasing rotation time, from 2 to 6 min, and then increased when further centrifugation was applied. Centrifugation led to accelerated sedimentation as well as particle flocculation. Flocculated particles did not successfully sediment at short centrifugation times and therefore act as a foulant in the upcoming filtration. Longer centrifugation times resulted in more foulant sedimentation and better polyphenol retention.

Polyphenol recovery (R_ph_) and fouling coefficient (k) were used as responses to the optimization of centrifugation conditions. Considering that the conditions to achieve the highest R_ph_ and the lowest k values were different, a compromise using a desirability function approach was made [19,20]. A detailed description of this method has been provided previously [21]. Briefly, an overall desirability function, which is a multiplicative model of individual desirability, was used. The optimal conditions, calculated from the models, correspond to a centrifugation speed of 4000 rpm (1195× *g*) and centrifugation duration of 7.8 min. The corresponding R_ph_ and k values were 34.5% and 29.5 m^−1^, respectively. The experiment was run under the optimal conditions and in triplicate. Results were not significantly different from the predicted ones, confirming the adequacy of the predicted models.

### 2.3. Anthocyanin Identification and HPLC-DAD-ESI-MS^2^ Profiles

**Figure 5:**
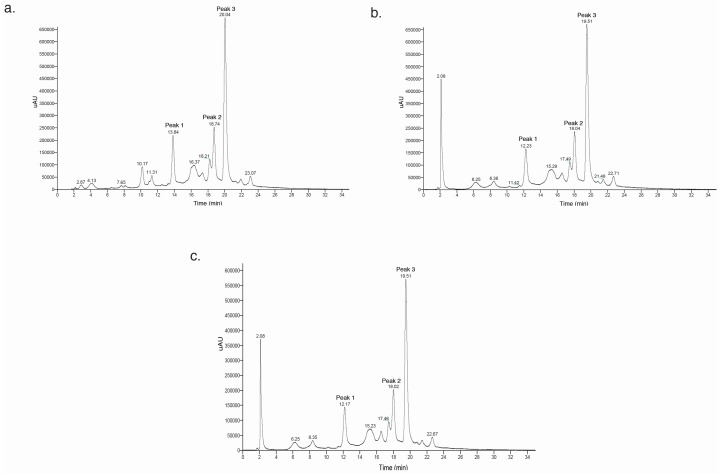
HPLC profiles of anthocyanins in (**a**) purple sweet potato (PSP) raw extract; (**b**) supernatant via centrifugation of raw extract at 4000 rpm for 7.8 min; and (**c**) permeate of supernatant via centrifugation and ultrafiltration (UF) (600 rpm, 30 kDa membrane, trans-membrane pressure (TMP) = 0.3 MPa).

In order to identify the main anthocyanins in the PSP extract and investigate the variation of anthocyanin content in the PSP extract before and after the centrifugation and filtration processes, HPLC-DAD-ESI-MS^2^ analyses were used to determine the anthocyanin profiles for PSP raw extract (S1), PSP juice after centrifugation at 4000 rpm for 7.8 min (S2) and the permeate of filtration of S2 with 30 kDa membrane under 0.3 MPa and a rotation speed of 600 rpm (S3). The HPLC-DAD-ESI-MS^2^ profiles are presented in Figure 5a–c. From the results obtained after HPLC-DAD-ESI-MS^2^ analysis, three main anthocyanin molecules were identified in the PSP extracts: (i) peonidin-3-feruloylsophoroside-5-glucoside (peak 1); (ii) peonidin-3-caffeoyl-*p*-hydroxybenzoylsophoroside-5-glucoside (peak 2) and (iii) peonidin-3-caffeoyl-feruloyl sophoroside-5-glucoside (peak 3), as previously reported [22,23,24,25,26] (Table 2). It should also be noted that the raw extract and post-centrifugation sample HPLC peak intensities (anthocyanins present in Figure 5) did not show variation, meaning that centrifugation is an efficient method for protein removal without anthocyanin loss. Moreover, the normalized peak area remained at ≈90% after filtration with 30 kDa membrane, showing slight anthocyanin loss. The removal of protein and other impurities by centrifugation might lead to a lesser fouling layer, which not only retains high molecular weight molecules, but also anthocyanins during the filtration process.

## 3. Materials and Methods

### 3.1. Samples

Purple sweet potatoes (PSP) were purchased from a local market in Wuhan, China. Fresh PSP samples (100 g each) were milled and accumulated using a Joyoung cooker (JYL-D022, Joyoung Corporation, Jinan, China) at a rotating speed of 20,000 rpm and a power of 250 W for extraction purposes.

### 3.2. PSP Extract Preparation

**Figure 6:**
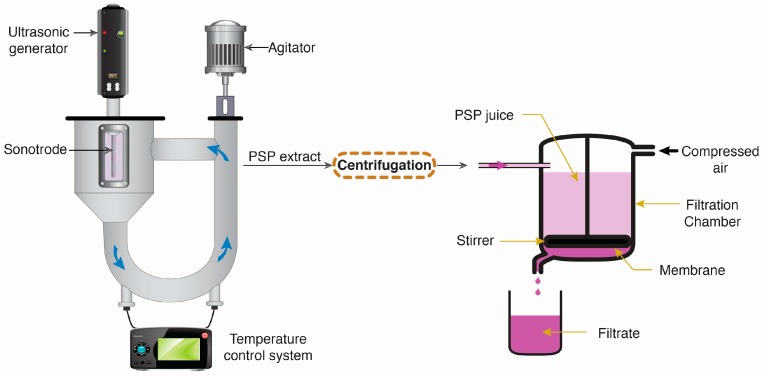
Schematic representation of ultrasonic circulating extraction equipment and ultrafiltration experimental set-up.

Ultrasonic circulating extraction equipment (Figure 6) (TGCXZ-10B, frequency 59 kHz, up to 1000 W power, Beijing Hong Xiang Long Co., Ltd., Beijing, China) equipped with an ultrasound horn-type probe of 20 mm diameter, was used for pilot scale extraction (500 g milled PSP sample). Twenty liters of deionized water were added as the extraction solvent. In this study, the ultrasonic treatment power was set at 840 W and the frequency was 59 kHz. The extraction temperature was fixed at 60 °C for 120 min. In the extraction test, a hydrochloric acid solution with a concentration of 4% (*v*/*v*) was added to give a pH ≈ 3 to the solvent, which is in the pH range for maximum anthocyanin color stability and thus prevents the degradation of these compounds [27]. The extract was pre-filtered on a mesh to remove the pulp, and then pooled and stored at −20 °C until needed for analysis.

### 3.3. Centrifugation of PSP Extract

Centrifugation of PSP extract was carried out in an Optima XE-100 ultracentrifuge (Beckman Coulter, Brea, CA, USA), in order to remove impurities, such as proteins and hydrocolloids that were present in the PSP extract. The centrifugation speed varied from 378 rpm (11× *g*) to 4621 rpm (1595× *g*) and the centrifugation duration varied from 0 to 6 min.

### 3.4. Filtration of PSP Juice

Polyphenol recovery and protein separation was performed via dead-end ultrafiltration (UF) coupled with rotation (Figure 6), in a stirred Amicon 8010 cell (effective membrane area of 4.1 × 10^−4^ m^2^ and maximal volume of 10 mL) (Millipore, Bedford, MA, USA). For each experiment, 10 mL of PSP juice was used and 6 mL of filtrate was obtained. Polyethersulfone UF membranes (Microdyn-Nadir GmbH, Wiesbaden, Germany) with molecular weight cut-offs (MWCO) of 30 kDa were used for all the filtration tests. New membranes were used for each set of experiments. The stirring was done using a magnetic stirrer fixed over the membrane surface and rotating at fixed rate (ω = 600 rpm). Ultrafiltration experiments were performed at room temperature by applying a trans-membrane pressure (TMP) of 0.3 MPa. The volume of filtrate obtained during filtration was collected and recorded.

### 3.5. Membrane Fouling Analysis with Exponential Model

In order to quantify membrane fouling, an exponential model was used for the analysis of filtration performance and fouling coefficient calculation. The exponential model proposed previously [28], and presented in Equation (5) assumes that the total resistance (R_tot_) to filtrate flow is empirically related to the filtrated volume:

(5) $$R_{\mathrm{tot}}={\;R}_{\mathrm{me}}\times e^{\frac{\mathrm{kV}}{A}},$$

where R_me_ is the membrane resistance (m^−1^) and k is the exponential fouling coefficient (m^−1^), which depends on many factors, including feed composition, operation conditions and membrane properties. V (m^3^) is the filtrate volume and A is the effective membrane area (m^2^).

Equation (6) can be obtained after rearrangement by substituting Equation (5) into the general equation of filtration, as previously reported [14]:

(6) $$V=\frac{A}{k}\ln(\frac{k\times P_{\mathrm{tm}}}{\mu \times R_{\mathrm{me}}}\times t+1),$$

where t is filtration time (s), μ is the dynamic viscosity (Pa·s) of the feed juice and P_tm_ is trans-membrane pressure (Pa).

By fitting the experimental data V(t) to this equation, the value of k, which can be used as a response for the centrifugation condition optimization, was determined.

### 3.6. Compound Analyses

#### 3.6.1. Polyphenol Analysis

##### HPLC-DAD-ESI-MS^2^ Anthocyanin Analysis

The anthocyanin profiles of the samples obtained from the PSP extract, centrifugation and filtration permeate were studied using HPLC-DAD-ESI-MS^2^, as recently reported [22]. An Accela series HPLC instrument (Thermo Fisher, San Jose, CA, USA) coupled with an Accela LTQ XL mass spectrometer (Thermo Fisher, San Jose, CA, USA) were used in this study. The HPLC instrument consisted of two Accela 600 pumps, Accela auto-sampler and Accela PDA detector. Chromatographic separation was carried out using a C18 reversed-phase column (250 mm × 4.6 mm, 5 μm, Merck, Billerica, MA, USA). The HPLC conditions were set as follows: column temperature was set at 25 °C, UV-Vis spectra were recorded in the wavelength range 220–780 nm, chromatograms were acquired on channel A (541 nm) and the injection volume was set at 10 μL. Prior to injection, all samples were filtered through a 0.2 μm cellulose acetate filter. The mobile phase was prepared with mixtures of formic acid/water (solvent A) and formic acid/acetonitrile/water (solvent B) at the ratios of 10/90 (volume of formic acid/volume of water), and 10/30/60 (volume of formic acid/volume of acetonitrile/volume of water), respectively. The elution gradient was as follows: 0 min: 20% (B), 70 min: 85% (B), 72 min: 100% (B), 75 min: 100% (B), 78 min: 20% (B), and 80 min: 20% (B) using a flow rate of 1 mL/min. MS conditions were as follows: sheath gas (N_2_) flow rate: 20 mL/min, spray voltage: 4.5 kV, capillary temperature: 270 °C, capillary voltage: 26 V and collision energy: 25–35V. Data acquisition was performed using X-Calibur software (version 2.1, Thermo fisher Scientific Inc., Waltham, MA, USA), and analyzed in positive spray ionization mode. A full scan MS-MS (MS^2^) mode of the most intense ions, determined using relative collision energy of 20 V, was applied.

##### Anthocyanin Analysis

Anthocyanin content (C_an_) was determined according to a pH-differential method based on the color change of anthocyanin with pH, as previously described [4]. The absorbance for each sample was measured at pH 1.0 and pH 4.5. The observed absorbance difference was proportional to the anthocyanin content, C_an_ (mg·L^−1^) and was calculated according to Equations (7) and (8):

(7) $$C_{\mathrm{an}}\left(\mathrm{mg}\cdot L^{-1}\right)=\;\frac{\mathrm{ABS}}{\varepsilon \times L}\times \;\mathrm{MW}\times \;D\times 10^{3},$$

(8) $$\mathrm{ABS}=\left(A_{541\mathrm{nm}}-A_{700\mathrm{nm}}\right){\mathrm{pH}}_{1.0}-\left(A_{541\mathrm{nm}}-A_{700\mathrm{nm}}\right){\mathrm{pH}}_{4.5},$$

where C_an_ is the total anthocyanin content, expressed as cyanidin-3-glucoside equivalent (CGE) (mg CGE/L), A_541_ and A_700_ are the absorbance values at 541 and 700 nm, respectively, MW is the molecular weight of cyanidin-3-glucoside (449.2 g·mol^−1^), D is the dilution factor, ε is the molar absorptivity of cyanidin-3-glucoside (26,900 L·mol^−1^·cm^−1^), L is the cell path length (1 cm in the present study) and 10^3^ is the conversion factor from g to mg.

Anthocyanin recovery (R_an_) was determined as expressed in Equation (9):

(9) $$R_{\mathrm{an}}\left(\%\right)=\frac{C_{\mathrm{an},\mathrm{permeate}}}{C_{\mathrm{an},\mathrm{feed}}},$$

where C_an,feed_ and C_an,permeate_ represent the concentrations of anthocyanin in the feed and the permeate solutions, respectively.

##### Total Phenolic Compounds

Polyphenol content (C_ph_) was determined using a Folin–Ciocalteu assay [29]. Standard solutions of gallic acid at different concentrations (0–0.1 mg·mL^−1^) were used for the calibration curve. One milliliter of sample, 1 mL Folin-Ciocalteu reagent and 1.5 mL 20% (*w*/*v*) Na_2_CO_3_ were added successively to a glass tube. The volume was then made up to 10 mL using distilled water. The solution was placed in the dark for 2 h at room temperature and then the absorbance was measured at 760 nm. C_ph_ was expressed as gallic acid equivalents (GAE) (mg GAE/L). Polyphenol recovery (R_ph_) was determined as expressed in Equation (10):

(10) $$R_{\mathrm{ph}}\left(\%\right)=\frac{C_{\mathrm{ph},\mathrm{feed}}}{C_{\mathrm{ph},\mathrm{permeate}}},$$

where C_ph,feed_ and C_ph,permeate_ (mg GAE/L) represent the concentrations of polyphenols in the feed and the permeate solutions, respectively.

#### 3.6.2. Total Protein Content

The protein concentrations (TC) in the extract and the permeate were determined as previously reported [30]. In brief, 1 mL of the sample was mixed with 5 mL of freshly prepared Coomassie Brilliant Blue G-250 solution in a glass tube. The volume was then adjusted to 10 mL. After 5 min incubation at room temperature, the absorbance was measured at 595 nm. Bovine Serum Albumin (BSA) was used for the calibration curve.

RC_pr_, representing the coefficients of protein rejection, was estimated using Equation (11):

(11) $${\mathrm{RC}}_{\mathrm{pr}}=\frac{C_{\mathrm{pr},\mathrm{permeate}}}{C_{\mathrm{pr},\mathrm{feed}}},$$

where C_pr,permeate_ and C_pr,feed_ are the protein concentrations in the filtrate and the feed, respectively.

### 3.7. Particle Size Distribution

The volume-based function of the particle size distribution SDF (%) of the PSP extract was measured using a Malvern Zen 3600 Zeta sizer instrument (Malvern Instrument, Malvern, UK). SDF was calculated using Zeta sizer software (Ver.7.11, Malvern Instruments, Malvern, UK).

### 3.8. Centrifugation Study by Experimental Design

Response surface methodology (RSM) using a Central Composite Design (CCD) was used to investigate the impact of two independent variables (centrifugation speed (X_1_), and centrifugation time (X_2_)), on polyphenol recovery and filterability.

Regression analysis was performed according to experimental data. RSM design and statistical analyses were performed using Design-Expert Version 7.0.0 software (Stat Ease Inc., Minneapolis, MN, USA). Coded and actual levels for the process of independent variables are shown in Table 3. The correspondence between coded and actual values can be obtained using the formula given in Equation (12):

(12) $$x_{i}=\frac{X_{i}-X_{i}^{0}}{{\Delta X}_{i}},$$

where x_i_ is the coded value, X_i_ is the corresponding actual value, $X_{i}^{0}$ is the actual value in the center of the domain and ∆X_i_ is the variation amplitude around the mean value.

Experimental data for polyphenol recovery and filterability were fitted to a quadratic model given by Equation (13):

(13) $$y=b_{0}+b_{1}x_{1}+b_{2}x_{2}+b_{12}x_{1}x_{2}+b_{11}x_{1}^{2}+b_{22}x_{2}^{2},$$

where x_1_ and x_2_ correspond to the coded independent variables, namely, centrifugation speed and centrifugation time, respectively. The b_n_ values represent the corresponding regression coefficients.

Five replicates at the center of the domain were used to estimate the pure error. The experiments were randomized in order to maximize the effects of unexplained variability in the observed responses.

The responses of each independent variable are listed in Table 4. The lack of fit was calculated in order to ensure satisfactory model optimization. The experimental Fisher value (*F*-value) is generally used to evaluate the significance of the model. Significance was tested at 95% confidence level.

## 4. Conclusions

The extraction of polyphenols from purple sweet potatoes has been carried out using ultrasonic circulating extraction equipment and water as a solvent. Ultrafiltration gave a polyphenol recovery of 23% and an anthocyanin recovery of 99%. Centrifugation pre-treatment of the PSP extract at 2500 rpm over 6 min increased the recovery of polyphenols to 29%. The positive effect of centrifugation on the filtration kinetics and membrane fouling has been demonstrated. By taking the polyphenol recovery and the fouling coefficient as responses, the centrifugation conditions were optimized by applying a response surface methodology using central composite design. Under the optimal conditions of 4000 rpm (1195× *g*) centrifugation speed and 7.8 min duration, the recovery of polyphenols and the fouling coefficient were of 34.5% and 29.5 m^−1^, respectively. Protein removal by centrifugation meant that the main anthocyanin content remained at ≈90% after filtration. This study demonstrates the promising potential of the ultrasound-assisted, combined green extraction, centrifugation and ultrafiltration process for the valorization of purple sweet potatoes.

## Figures and Tables

**Table 1:** Analysis of variance (ANOVA) for the response surface methodology (RSM) models of polyphenol recovery and the fouling coefficient.

Source	Sum of Squares	df	Mean Square	*F*-Value	*p*-Value (Prob > *F*)	
Polyphenol recovery ^a^						
Model	135.09	5	27.02	4.62	0.0349	Significant
Residual	40.94	7	5.85			
Lack of fit	32.64	3	10.88	5.24	0.0718	Not significant
Pure error	8.3	4	2.08			
Fouling coefficient ^b^						
Model	262.73	5	52.55	4.85	0.0309	Significant
Residual	75.77	7	10.82			
Lack of fit	61.83	3	20.61	5.91	0.0594	Not significant
Pure error	13.94	4	3.49			

Note: df denotes degree of freedom. ^a^ R^2^ = 0.8; ^b^ R^2^ = 0.8.

**Table 2:** Anthocyanin identification of purple sweet potato (PSP) extracts and samples obtained via the centrifugation and filtration processes.

Peak	*m*/*z*		Anthocyanins	Normalized Peak Area		
	[M]^+^	Fragment Ions		S1	S2	S3
1	963	801; 463; 301	Peonidin 3-feruloyl sophoroside-5-glucoside	1	1	0.88
2	1069	907; 463; 301	Peonidin 3-caffeoyl-*p*-hydroxybenzoyl sophoroside-5-glucoside	1	1	0.96
3	1125	963; 463; 301	Peonidin 3-caffeoyl-feruloyl sophoroside-5-glucoside	1	1	0.86

S1: Raw extract; S2: Sample obtained after centrifugation at 2500 rpm and 6 min; S3: Sample obtained from filtration of sample 2 with 30 kDa membrane under 0.3 MPa and 600 rpm.

**Table 3:** Independent variable values of the centrifugation process and their corresponding levels.

Independent Variables	Symbol		Levels		
	Actual	Coded	−1	0	1
Speed (rpm)	X_1_	x_1_	1000	2500	4000
			(75× *g*)	(467× *g*)	(1195× *g*)
Time (min)	X_2_	x_2_	2	6	10

**Table 4:** RSM design and its experimental values.

Run	Independent Variables		Response Variables	
	Centrifugation Speed (rpm) X_1_(x_1_)	Centrifugation Time (min) X_2_(x_2_)	Polyphenol Recovery (%)	Fouling Coefficient (m^−1^)
1	2500 (0)	6 (0)	29	25.78
2	2500 (0)	6 (0)	31	25.67
3	2500 (0)	6 (0)	27.3	25.39
4	1000 (−1)	2 (−1)	31.3	39.48
5	2500 (−1)	0.3 (−1.414)	37	38.05
6	4621 (+1.414)	6 (0)	38.1	33.67
7	379 (−1.414)	6 (0)	29.8	31.71
8	2500 (0)	12 (+1.414)	33.5	37.45
9	4000 (+1)	2 (−1)	29.6	27.34
10	1000 (−1)	10 (+1)	27.5	39.07
11	2500 (0)	6 (0)	27.8	29.45
12	2500 (0)	6 (0)	28.3	28.45
13	4000 (+1)	10 (+1)	36.8	32.62
